# Genome-wide identification and characterization of NPF family reveals NtNPF6.13 involving in salt stress in *Nicotiana tabacum*

**DOI:** 10.3389/fpls.2022.999403

**Published:** 2022-10-13

**Authors:** Hui Zhang, Zefeng Li, Guoyun Xu, Ge Bai, Peipei Zhang, Niu Zhai, Qingxia Zheng, Qiansi Chen, Pingping Liu, Lifeng Jin, Huina Zhou

**Affiliations:** ^1^ China Tobacco Gene Research Center, Zhengzhou Tobacco Research Institute of China National Tobacco Corporation (CNTC), Zhengzhou, China; ^2^ National Tobacco Genetic Engineering Research Center, Yunnan Academy of Tobacco Agricultural Sciences, Kunming, China

**Keywords:** *Nicotiana tabacum*, nitrate transporter 1/peptide transporter family, NPF, salt stress, gene expression, chloride

## Abstract

Proteins of the Nitrate Transporter 1/Peptide Transporter (NPF) family transport a diverse variety of substrates, such as nitrate, peptides, hormones and chloride. In this study, a systematic analysis of the tobacco (*Nicotiana tabacum*) *NPF* family was performed in the cultivated ‘K326’. In total, 143 *NtNPF* genes were identified and phylogenetically classified into eight subfamilies, NPF1 to NPF8, based on the classification of NPF families in other plant species. The chromosomal locations and structures of the *NtNPF* genes were analyzed. The expression profiles of *NtNPF* genes under NaCl stress were analyzed to screen the possible *NPF* genes involving in chloride regulation in tobacco. Most *NtNPF6* genes responded to salt stress in the roots and leaves. The expression of *NtNPF6.13* was significantly down-regulated after salt stress for 12h. The chloride content was reduced in the roots of *ntnpf6.13* mutant. These findings support the participation of *NtNPF6.13* in chloride uptake. Several other *NtNPF* genes that play potential roles in chloride metabolism of tobacco require further study.

## Introduction

As an important macronutrient, nitrogen (N) plays an essential role in plant growth and development (Crawford, 1995; Xu et al., 2012). Among the different forms of nitrogen, nitrate ( $NO_{3}^{-}$ ) is predominant in plant roots. The four most reported nitrate transport protein families are the Nitrate Transporter 1/Peptide Transporter family (NPF), the Nitrate Transporter 2 family (NRT2), the Chloride Channel family (CLC), and the slow anion channel-associated homologues family (SLAC/SLAH) (Krapp et al., 2014).The NPF family typically comprises a large number of members within a species, for example, 53 NPFs in Arabidopsis and 93 NPFs in rice are known and have been well studied (Leran et al., 2014; Wang et al., 2018b). The NPF proteins can be classified into eight subfamilies and named by a standard rule. In Arabidopsis, 20 *AtNPF* genes are involved in nitrate uptake or efflux from the soil. AtNPF6.3, also named *NRT1.1* or *CHL1*, was the first nitrate transporter to be cloned and has dual-affinity nitrate transport activity (Tsay et al., 1993). Regardless of their participation in nitrate uptake, root-to-shoot transport, or leaf nitrate allocation, *AtNPF* genes all have important functions. In other species, *NPF* genes have been reported to contribute to nitrate transportation, for example, *NPF2.2* in rice (Li et al., 2015), *NPF6* in maize (Wen et al., 2017), and *NPF6.5* in grape (He et al., 2020).

In addition to nitrate transport, NPF proteins perform diverse functions (Anfang and Shani, 2021; Prabhala et al., 2021).An increasing number of other substrates of NPF proteins have been reported (Corratge-Faillie and Lacombe, 2017; Payne et al., 2017; Wulff et al., 2019), of which one is chloride. It is well known that CLC and SLAC/SLAH genes encode chloride transporters or channels that participate in chloride transport (Barbier-Brygoo et al., 2011; Zhang et al., 2018).The micronutrient Cl^-^ shows strongly dynamic interaction with $NO_{3}^{-}$ (Wege et al., 2017). Given the similar physical properties in solution, the selectivity of proteins for these two monovalent anions is often ambiguous. In Arabidopsis, two *NPF* members are involved in chloride transport, namely *AtNPF2.4* (Li et al., 2016a), and *AtNPF2.5* (Li et al., 2016b). *AtNPF2.4*is highly expressed in the root stele and facilitates the transfer of chloride from root to the shoot. Accumulation of Cl^-^ increases with overexpression of *AtNPF2.4*and is reduced with knockdown of *AtNPF2.4*. *AtNPF2.5*, which is the closest homolog to *AtNPF2.4*, is expressed predominantly in the root and modulates chloride efflux from the root. Both *AtNPF2.4* and *AtNPF2.5*may modulate Cl^-^ transportation without affecting $NO_{3}^{-}$ accumulation in the shoot. In maize, ZmNPF6.4 and ZmNPF6.6 transport both chloride and nitrate (Wen et al., 2017). With regard to chloride transport, ZmNPF6.4 shows high-affinity chloride transport activity, whereas ZmNPF6.6 shows low-affinity chloride transport activity. In contrast, with respect to nitrate transport, ZmNPF6.4 exhibits low-affinity nitrate transport activity, whereas ZmNPF6.6 shows high-affinity nitrate transport activity. Recently, it has been reported that *MtNPF6.5* and *MtNPF6.7* mediate chloride uptake and nitrate preference in *Medicago* roots (Xiao et al., 2021). Apart from these reported *NPF* genes, little information is available on the participation of *NPF* genes in chloride transport in other species.

To date, the *NPF* gene family has been identified and characterized in many plant species, including Arabidopsis (Chiba et al., 2015), wheat (Wang et al., 2020), rice (Drechsler et al., 2018), apple (Wang et al., 2018a), rapeseed (Zhang et al., 2020), spinach (Wang et al., 2021), potato (Zhang et al., 2021) and poplar (Zhao et al., 2021). No previous study has investigated the *NPF* gene family in *Nicotiana* genus. In the current study, we identified the *NPF* family members of tobacco (*Nicotiana tabacum)* and analyzed the phylogenetic relationships, gene structure and chromosomal location. To screen *NtNPF* genes involved in chloride transportation, the expression profiles of *NtNPF* genes were evaluated using an RNA sequencing (RNA-seq) data from plants exposed to salt stress. The results revealed that 80% of *NtNPF6*genes were responsive to salt stress either in the roots or the leaves. Using the CRISPR/Cas9 gene-editing system to knockout *NtNPF6.13*, one of most responsive *NtNPF6* members in roots under salt stress, the chloride content of roots was significantly decreased in the mutants compared to the wild type. The findings indicate that *NtNPF6.13* might play an important role in chloride uptake.

## Materials and methods

### Identification of *NPF* genes in tobacco

Genome sequences of ‘K326’ (a commonly cultivated tobacco cultivar) were downloaded from the Sol Genomics Network (https://solgenomics.net/). Fifty-three AtNPF seqences were obtained from The Arabidopsis Information Resource database (https://www.arabidopsis.org/). The AtNPF protein sequences were used to perform a BLASTP search in the K326 database with E-value<1e-5. The PFAM domain (PF00854.18: PTR2) was used to identify NtNPFs. The online ExPASy tool (https://www.expasy.org/) was used to predict the molecular weights and isoelectric points of each NtNPF protein.

### Phylogenetic analysis of NtNPF proteins

Protein sequence alignments for Arabidopsis (53 AtNPFs) and tobacco (143 NtNPFs) NPF family members were generated using ClustalW (https://www.genome.jp/tools-bin/clustalw). A phylogenetic tree was constructed with RAxML (version 8.2.10) under the PROTGAMMAGTR model with 100 bootstrap replications. The phylogenetic tree was edited and visualized in Evolview V3 (https://www.evolgenius.info/evolview/) (Subramanian et al., 2019).

### Chromosomal localization and syntenic analysis of *NtNPF* genes

The chromosomal location of the *NtNPF* genes was determined from the Sol Genomics Network database. Syntenic blocks were identified using MCScanX (Wang et al., 2012). The results were visualized with Circos software (Krzywinski et al., 2009), with syntenic blocks involving *NtNPF* gene pairs connected by lines.

### Gene structure and conserved motifs analysis of *NtNPF* gene family

The genomic DNA and CDS information for the *NtNPF* genes were downloaded from Sol Genomics Network database. To explore the structure of *NtNPF* genes, the Gene Structure Display Server 2.0online tool (GSDS, http://gsds.gao-lab.org/index.php; Hu et al., 2015) was used to display the genomic length and organization of introns/exons. The conserved motifs of NtNPFs were analyzed using the MEME online tool (version 5.3.0, http://meme-suite.org/tools/meme; Bailey et al., 2015). The maximum number of motifs was set 10.

### Cis-regulatory element analysis of *NtNPFs*

To predict the function of *NtNPFs*, putative cis-reguatory elements were analyzed. The 2-kb upstream sequence from the translation start site of all 143 *NtNPF* genes was obtained and set as the promoter. The sequences were analyzed using the plantCARE database (http://bioinformatics.psb.ugent.be/webtools/plantcare/html/; Lescot et al., 2002).

### Plant materials and salt treatment

Seedlings of tobacco’K326’ were cultivated in plastic pots under a 16-h photoperiod at 28 and 23 °C (day/night). For salt treatment, plants at the six-leaf stage were transferred to a nutrient solution for 1 week, and then NaCl (final concentration 300 mM) was added to the solution to initiate salt treatment. After treatment for 12 h, 3d or 7 d, leaves and roots were sampled and used for RNA extraction and RNA-seq. Three biological replicates were used and the nutrient solution without NaCl was used as control (CK). All samples were immediately frozen in liquid nitrogen and stored at -80 °C, except that the roots were washed with water before freezing. The nutrient solution contains 5mM potassium nitrate (KNO_3_), 1 mM magnesium sulfate heptahydrate (MgSO_4_·7H_2_O), 1 mM monopotassium phosphate (KH_2_PO_4_), 4 mM calcium nitrate tetrahydrate (Ca(NO_3_)_2_·4H_2_O), 1mM ammonium nitrate (NH_4_NO_3_), 0.1 mM ferric sodium EDTA (Fe-Na-EDTA), 0.1mM boric acid (H_3_BO_3_), 30 µM zinc sulfate (ZnSO_4_·7H_2_O), 100 µM manganese monosulfate (MnSO_4_·H_2_O), 0.1 µM copper sulfate pentahydrate (CuSO_4_·5H_2_O), 0.1 µM cobalt chloride hexahydrate (CoCl_2_·6H_2_O) and 1 µM sodium molybdate dihydrate (Na_2_MoO_4_·2H_2_O) per liter, with about 0.2 μM of background chloride concentration.

### RNA isolation and qPCR analysis

Total RNA was isolated using the RNAprep Pure Plant Kit (Tiangen, Beijing, China) in accordance with the manufacturer’s protocol. The RNA quality and concentration were determined using a Nano Drop 2000 spectrophotometer. The cDNA was synthesized using the Transcriptor First Strand cDNA Synthesis Kit (Roche). Quantitative real-time PCR (qPCR) analysis was performed as described previously (Zhang et al., 2018). All specific primers used are listed in **Supplementary Table S1**.

### RNA-sequencing and transcriptomic analysis

For RNA-Seq, total RNA was extracted from the K326 root and leaf samples (CK and treated with 300mM NaCl for 12h, 3d or 7d) with the RNAprep Pure Plant Kit. Each sample, comprising three seedlings, was analyzed in triplicate under the same conditions. A total of 24 samples were used for RNA-seq by the Novogene Company (Beijing, China) on an Illumina HiSeq4000 platform. After filtering for quality control, the clean reads were mapped to the K326 genome (ftp://ftp.solgenomics.net/genomes/Nicotiana_tabacum/edwards_et_al_2017/assembly/) with Hisat2 (Kim et al., 2019). Gene abundances were estimated using StringTie (Pertea et al., 2015). The expression levels of *NtNPF* genes were then analyzed.

### Subcellular localization of NtNPF6.13

The subcellular localization of NtNPF6.13 was first predicated with the online tools Plant-mPLoc (http://www.csbio.sjtu.edu.cn/bioinf/plant-multi/; Chou and Shen, 2010), WoLF PSORT (https://wolfpsort.hgc.jp; Horton et al., 2007), YLoc (https://abi-services.informatik.uni-tuebingen.de/yloc/webloc.cgi; Briesemeister et al., 2010) and PSORT (https://www.genscript.com/tools/psort; Nakai and Horton, 1999). The online tool TMHMM2.0 (https://services.healthtech.dtu.dk/service.php?TMHMM-2.0) was used to predict the transmembrane helices of NtNPF6.13.To verify the predicted localization, *NtNPF6.13* was cloned without the termination codon using a pair of gene-specific primers, (5’-ATGGCACTTCCTGAGACAC-3’ and 5’-ACAAACCGGTCCATCATC-3’). The fragment was purified and inserted into the vector pFFCS1300 to fuse the C-terminus with the green fluorescent protein (GFP) under the constitutive control of the CaMV35S promoter. After sequencing, the recombinant plasmid 35S: *NtNPF6.13-GFP* was transferred to *Agrobacterium tumefaciens* strain GV3101. The fusion construct was infected into the leaf of *Nicotiana benthamiana* and the GFP signal was observed using a TCS STED CW confocal laser microscope (LEICA, Wetzlar, Germany).

### Generation of *NtNPF6.13* knockout lines and salt treatment

The CRISPR/Cas9-based genome editing method was used to generate*NtNPF6.13* knockout lines. A 20-bp coding sequence (5’-GCTCTTAGATCCTCCTCCGG-3’) of *NtNPF6.13* was inserted into the sgRNA-Cas9 expression vector and transformed into tobacco K326. A 252-bp DNA fragment was amplified from the transgenic lines by using a primer pair (5’-CTTCCTGAGACACAGCAAG-3’ and 5’-TTCCCATCTAATCTCGCC-3’) bordering the target region. The PCR products were sequenced to determine the mutation sites of *NtNPF6.13*. The existence of Cas9 sequence in T_1_ transgenic plants was examined using a Cas9-sepecific primer pair (5’-GGGACCCTAAGAAGTACGGC-3’ and 5’-TATTCTCGGCCTGCTCTCTG-3’).Plants free of Cas9 were retained and the*NtNPF6.13* genotype was determined.

Using the NaCl treatment method described above, *ntnpf6.13* mutants and K326 control plants were treated with 300 mM NaCl for 7 d. The roots were collected for ion determination. For phenotyping of plants under the salt stress, *ntnpf6.13* mutants and K326seeds were geminated on 1/2 MS plates for 1 week, then the seedlings were transferred to fresh MS plates supplemented with 150 mM NaCl for additional1 week of growth before photographing and measurement of the root length.

### Determination of chloride content in roots

The concentration of Cl^-^ was determined as described previously (Zhang et al., 2018). Briefly, the roots were dried and ground into powder. Then, 50 mg powder was digested with 10 ml of 5% acetic acid. After incubation at 30 °C for 30min, the digested solution was filtered and diluted, and analyzed with an AA3 Continuous Flow Analytical System (Germany).

## Results

### Genome-wide identification and characterization of tobacco *NPF* genes

To comprehensively identify the *NtNPF* genes in tobacco, NPF protein sequences of Arabidopsis were used to perform a BLASTP search of the genome database of *Nicotiana tabacum* (K326) from Sol Genomics Network. As shown in **Supplementary Table S2**, 143 *NtNPF* genes were identified and named in accordance with the nomenclatural rules for the gene family (Leran et al., 2014). The gene sequence length ranged from 375bp (*NtNPF4.18*) to 18,162bp (*NtNPF7.10*). The corresponding proteins were predicted ranging from 103aa (NtNPF2.8) to 1,158aa (NtNPF7.10). The length of more than 65% of NtNPF proteins was 500 aa -600 aa. The isoelectric point ranged from 4.31 (NtNPF5.9) to 10.99 (NtNPF2.15), and in most NtNPFs (>85%) was greater than7.

### Phylogenetic tree of *NtNPF* gene family in tobacco

**Figure 1:**
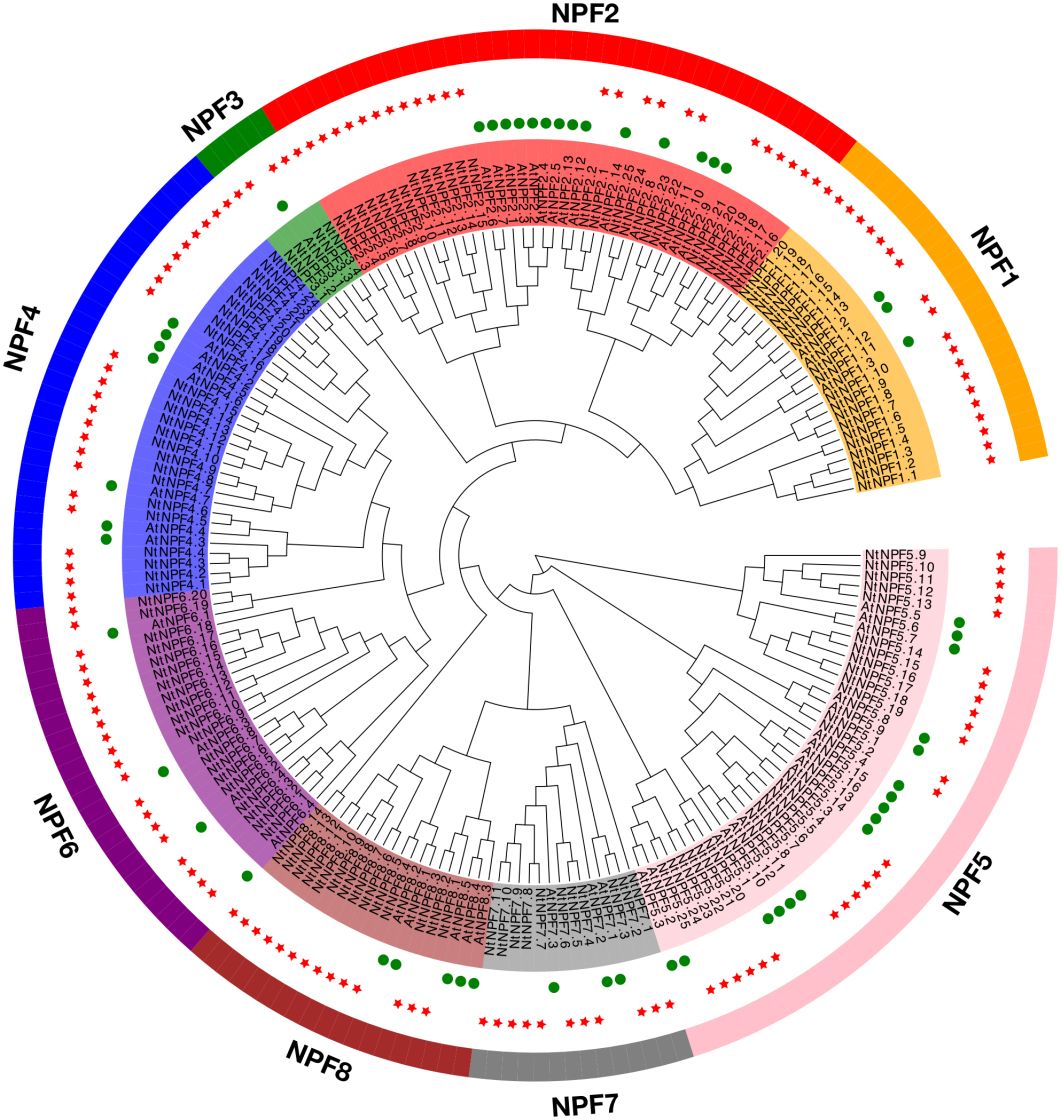
Phylogenetic tree of *NtNPF* genes of tobacco and *AtNPF* genes of Arabidopsis. The clades representing different subfamilies (groups 1–8) are indicated by different colors. *NtNPF* genes are marked with a green circle and *AtNPF* genes are marked with a red star.

To gain insight into the phylogenetic relationship of the 143NtNPFs, a phylogenetic tree was constructed using RAxML version 8.2.10 under the PROTGAMMAGTR model together with 53 AtNPF proteins. Based on the relationships with AtNPFs, the NtNPFs were classified into eight subfamilies (**Figure 1**), which was consistent with the classifications of NPFs from other species, such as *Brassica napus* (Wen et al., 2020), apple (*Malus × domestica Borkh.*) (Wang et al., 2018a), and spinach (Wang et al., 2021). The eight subfamilies were designatedNtNPF1 to NtNPF8, and each contained 20, 25, 4, 24, 25, 20, 11 and 14 members, respectively.

### Chromosomal locations and synteny analysis of *NtNPF* genes

**Figure 2:**
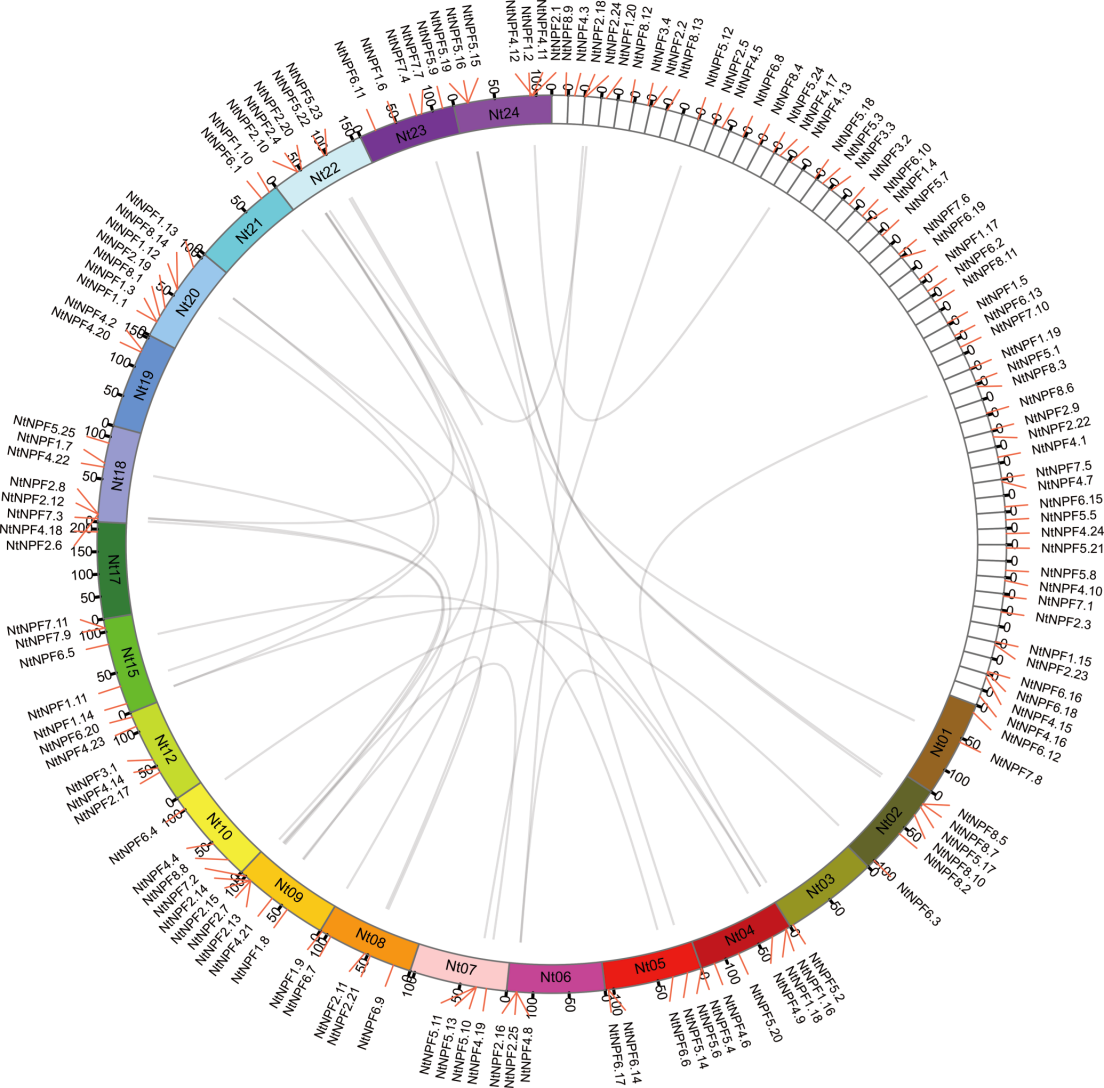
Chromosomal distribution and segmental duplication of *NtNPF* genes in tobacco. The panel shows the 20 chromosomes and unassigned scaffolds using a circle; gray lines connect homologous genes.

The chromosomal distribution showed that 88 of the 143 *NtNPF* genes were mapped on 20 of 24 tobacco chromosomes (**Figure 2**). Five chromosomes (Nt04, Nt09, Nt18, Nt20 and Nt24) carried seven *NtNPF* genes each, one chromosome (Nt02) carried six *NtNPF* genes, five chromosomes (Nt08, Nt12, Nt15, Nt22 and Nt23) harbored five *NtNPF* genes each, two chromosomes (Nt05 and Nt07) contained four *NtNPF* genes each, two chromosomes (Nt06 and Nt10)carried three genes each, three chromosomes (Nt01, Nt19 and Nt21) harbored two *NtNPF* genes each, and two chromosomes (Nt03 and Nt17) each carried a single *NtNPF* gene. With regard to the four *NtNPF3* genes, only one was mapped to a chromosome; the remaining three were mapped to scaffolds. In addition, it was noted that most *NtNPF* genes were located on the chromosome arms, whereas several *NtNPF* genes, such as *NtNPF5.4* and *NtNPF5.6*, were positioned near the telomere.

Using the predicted NtNPF protein sequences, a snyteny analysis was performed with MCScanX. Thirty-nine *NtNPF* genes were included in syntenic blocks, forming 26 syntenic *NtNPF* gene pairs (**Figure 2**). The 39 *NtNPF* genes belonged to seven *NtNPF* subfamilies excluding the *NtNPF3* subfamily. The *NtNPF5* subfamily contributing nine genes, *NtNPF1*, *NtNPF2*, and *NtNPF4* contributed seven genes each, *NtNPF6* and *NtNPF7* contributed four genes each, and*NtNPF8* contributed one gene.

### Gene structure and conserved motifs of *NtNPF* genes in tobacco

**Figure 3:**
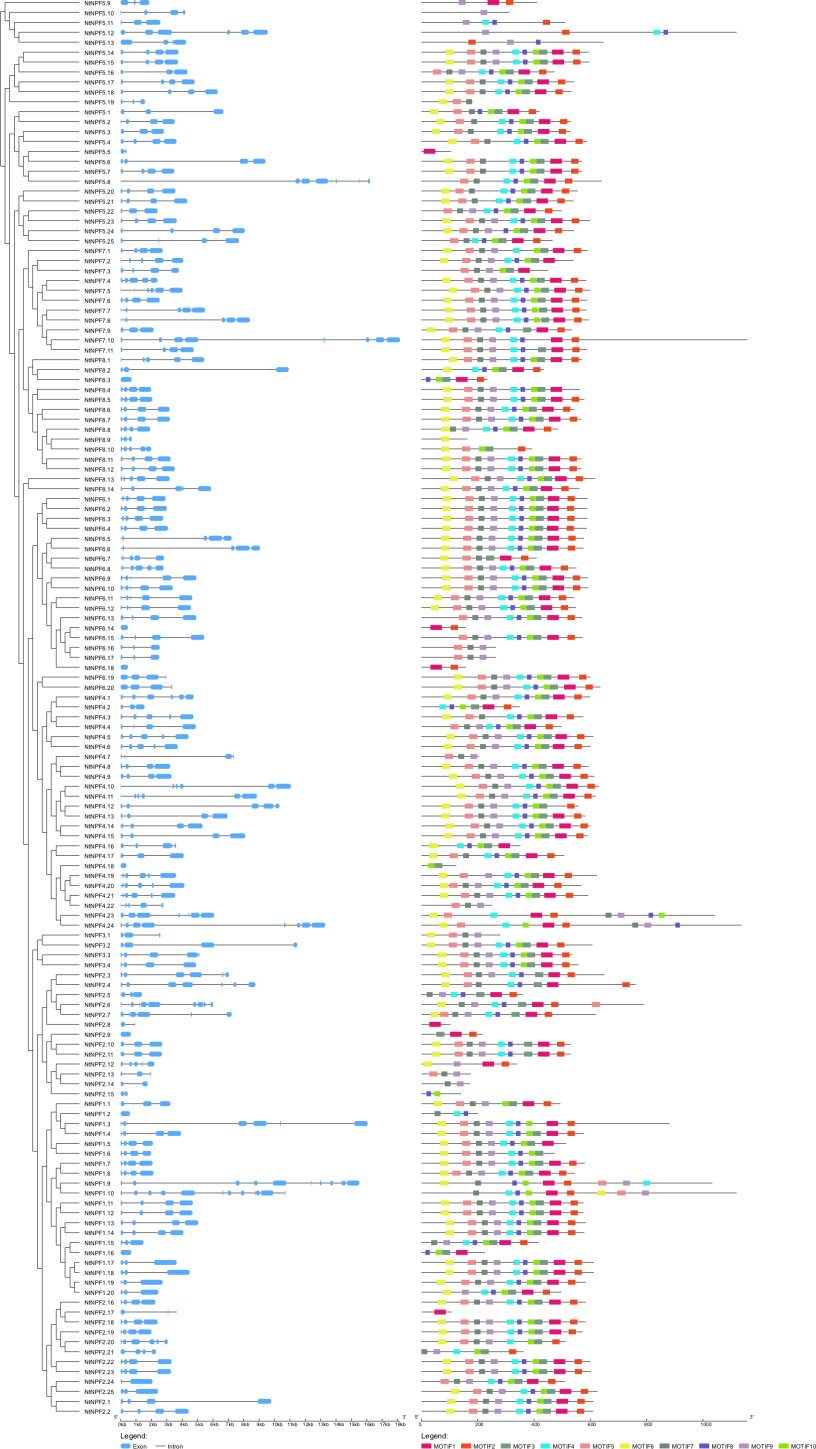
Phylogenetic relationships, gene structure, and architecture of conserved protein motifs of *NtNPF* genes in tobacco.

The *NtNPF* gene structure was determined using the GSDS online tools. The exon number of the 143 *NtNPFs* ranged from 1 to 13, and almost 110*NtNPFs* contained 3-5 exons (**Figure 3**). The longest *NtNPF* gene, *NtNPF7.10*, contained eight exons, whereas*NtNPF1.10*included the highest exon number (13) among all *NtNPF* genes. Seven genes, including*NtNPF1.2*, *NtNPF1.16* and *NtNPF2.9*, contained no introns. The diversity of the gene structure might imply an abundance of gene functions. Using the multiple sequence alignment of tobacco NPF proteins, the MEME tool was used to investigate the sequence features and functional motifs. Ten conserved motifs for NtNPF proteins were identified, which were designated motif 1 to motif 10 (**Figure 3**).

### Cis-acting elements in the promoters of *NtNPF* genes

**Figure 4:**
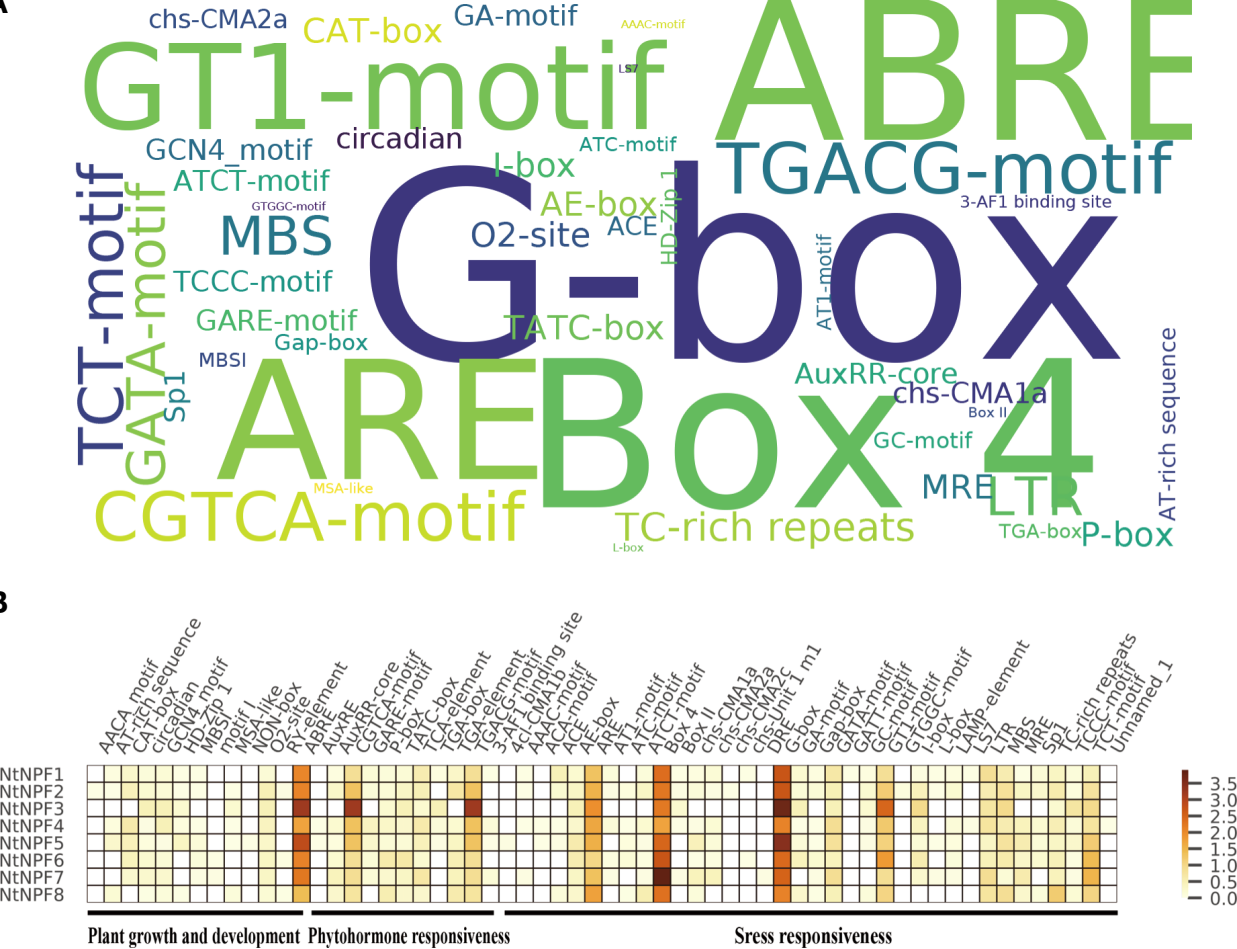
*Cis*-acting elements in the promoter of *NtNPF* genes of tobacco. **(A)** Over-presentation of the *cis*-acting elements in the promoter of all *NtNPF* genes. The font size is positively associated with the occurrence number of corresponding *cis*-acting elements. **(B)** Heatmap of average number of *cis*-acting elements in different *NtNPF* subfamilies. Brown represents a high number and white represents a low number of *cis*-acting elements.

The promoter (2 kb upstream) of each gene was analyzed using the PlantCARE database to explore the cis-acting regulatory elements of the *NtNPF* genes. Multiple cis-elements in the *NtNPF* gene promoters were detected, such as plant hormone response elements (auxin, methyl jasmonate [MeJA], gibberellin, salicylic acid and abscisic acid), abiotic stress response elements (light, low-temperature, wound, hypoxia and drought), biotic stress response elements and circadian control elements (**Figure 4**).The G-box, Box 4, ABRE and ARE elements were most frequently enriched in the *NtNPF* promoter regions (**Figure 4A** and **Supplementary Table S3** & **Figure S1**). The CGTCA-motif, TGACG-motif and GT1-motif elements, which are associated with MeJA response, were mostly enriched in the promoters of *NtNPF3* subfamily genes (**Figure 4B**), which indicated that *NtNPF3*genes might function in MeJA regulation.

### Transcriptional profiles of *NtNPF* genes under salt stress

**Figure 5:**
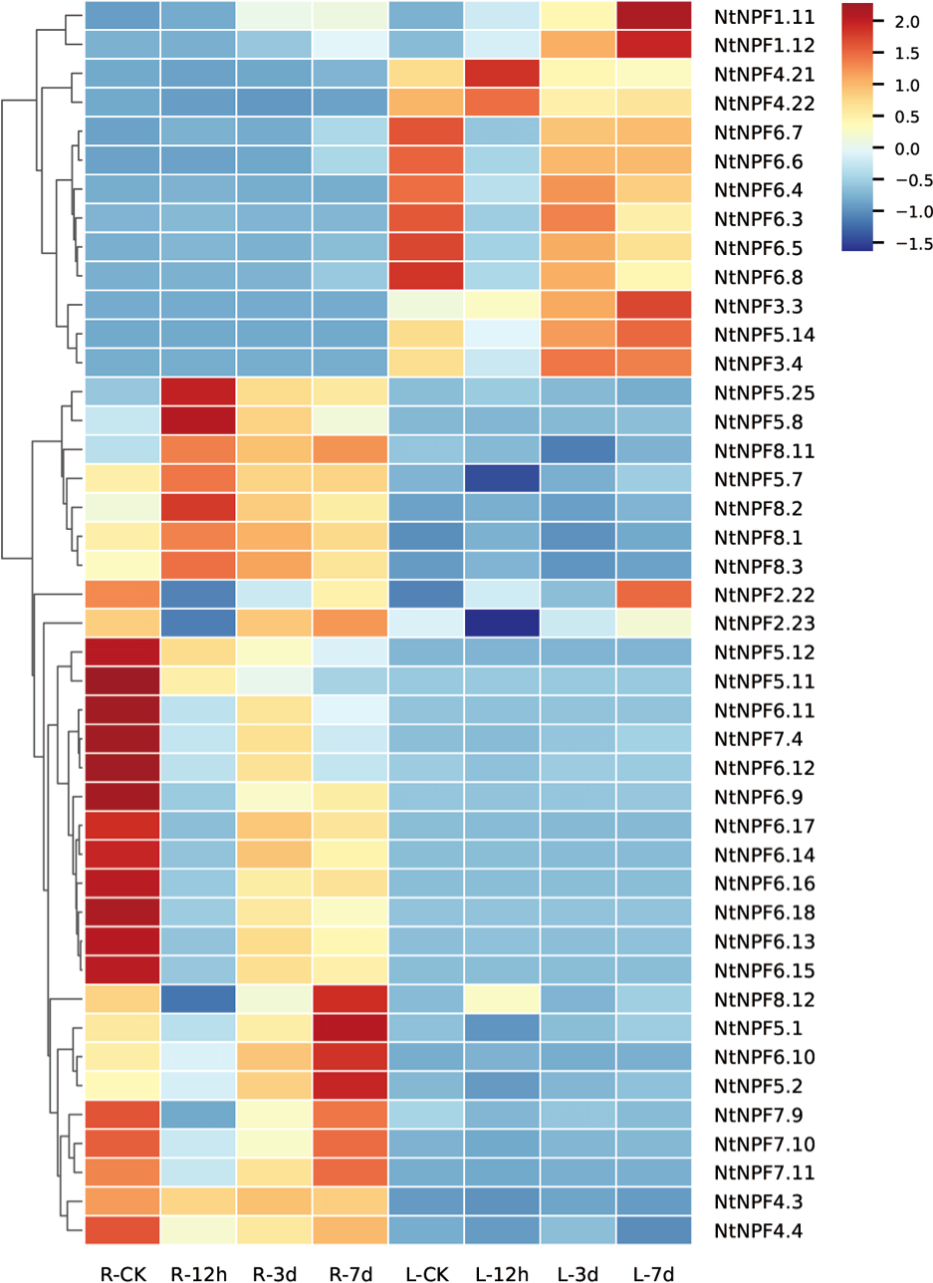
Expression profiles of *NtNPF* genes responsive to salt stress in roots and leaves of tobacco. R-CK, R-12h, R-3d and R-7d represent root samples, and L-CK, L-12h, L-3d and L-7d represent leaf samples. The expression values are shown as the *z* score of the RPKM values. The scale bar is shown on the right, and high expression levels are indicated by a red color.

In order to screen the potential *NtNPF* genes in response to salt stress, the transcriptional profile was analyzed using RNA-seq data sets for the roots and leaves derived from tobacco K326 seedlings exposed to 300mM NaCl stress for 0h, 12h, 3d and 7d.Among the 143 *NtNPF* genes, more than 70% showed extremely low or undetectable transcript levels before and after exposure to salt stress (**Supplementary Figure S2**).In contrast, several *NtNPF6*genes, namely*NtNPF6.16*, *NtNPF6.18*, *NtNPF6.13*, *NtNPF6.12*, and *NtNPF6.11*, showed the highest expression levels in the root prior to salt stress (**Figure 5** and **Supplementary Table S4**). Thirteen *NtNPF* genes responded to salt stress in the leaves and 30 *NtNPF* genes responded to salt stress in the roots. Two *NtNPF* genes, *NtNPF2.22* and *NtNPF2.23*, responded to salt stress in the roots and leaves. Almost all *NtNPF6*subfamily members responded to salt stress either in the leaves or the roots, except *NtNPF6.1*, *NtNPF6.2*, *NtNPF6.19* and *NtNPF6.20*. Interestingly, *AtNPF6.3* homologous *NtNPF6* genes usually responded in the roots, whereas*AtNPF6.2* homologous *NtNPF6* genes usually responded in the leaves. Notably, most of the responsive *NtNPF* genes were down-regulated at 12h under salt stress both in the roots and leaves. The root-specific highly expressed *NtNPF6.13*, *NtNPF6.16* and *NtNPF6.18* showed strongly similar expression patterns in response to salt stress: transcription was down-regulated after salt stress for 12h; and thereafter was mostly recovered at 3d and 7d under salt stress. The expression changes of the aforementioned *NtNPF* genes indicated that these *NtNPF* genes might play important roles in tolerance to salt stress in tobacco.

Additionally, the expression levels of 16 selected *NtNPF6* genes, *NtNPF6.3* to *NtNPF6.18*, were evaluated by qRT-PCR. Two distinct expression patterns were identified within 7 d of salt treatment (**Supplementary Figure S3**), except for *NtNPF6.5*, which was not detected in all samples. *NtNPF6.4*was the only gene that was initially up-regulated, and thereafter the expression level continuously decreased with prolonged salt treatment. The expression levels of the remaining 14 *NtNPF6* genes showed a similar pattern with the duration of salt treatment, namely significantly reduced expression after salt stress for 12 h, and thereafter recovery to different extents at 3 d of salt treatment. These results were essentially consistent with the transcriptome data. It was noted that *NtNPF6.13*, a highly expressed gene in the root, showed the greatest decline in expression level at 12hof salt treatment and recovered the least at 3d of salt treatment. Therefore, *NtNPF6.13* was selected to detect its function in response to salt stress.

### Subcellular localization of NtNPF6.13

**Figure 6:**
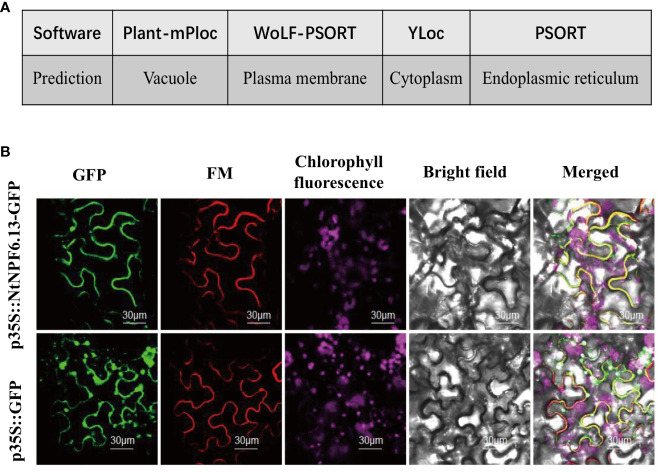
Subcellular localization of NtNPF6.13. **(A)** Localization predicted by different online tools. **(B)** Co-localization of NtNPF6.13-GFP with the plasma membrane marker FM in *N.benthamiana* leaves. Confocal images were captured the day after agroinfiltration. NtNPF6.13-GFP represents the gene and GFP represents the control with an empty vector.

The NtNPF6.13 protein was inconsistently predicted to be localized in the vacuole, plasma membrane, cytoplasm or endoplasmic reticulum, respectively, by four online tools (**Figure 6A**). To clarify its subcellular localization, *NtNPF6.13*fused with GFP was transformed in *Agrobacterium* and inoculated in *N. benthamiana* leaves. As shown in **Figure 6B**, NtNPF6.13 protein co-localized with the plasma membrane marker FM4-64 (Bolte et al., 2004), which indicated that NtNPF6.13 was localized to the plasma membrane. This result is consistent with the prediction by WoLF PSORT and the predicted existence of transmembrane region by TMHMM.

### Knockout of *NtNPF6.13* reduces Cl^-^ content in root

**Figure 7:**
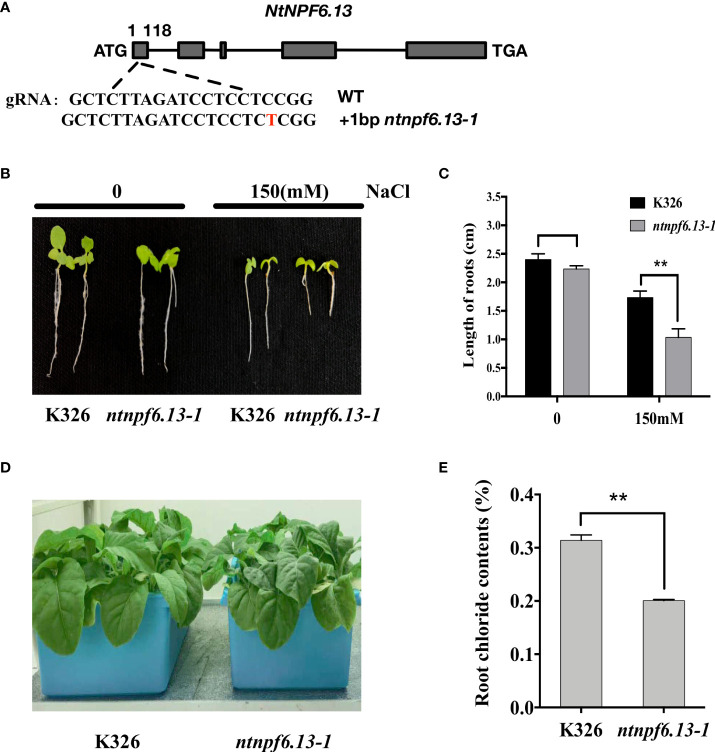
CRISPR/Cas9-mediated knockout of *NtNPF6.13* reduces the Cl^−^ content in roots of tobacco. **(A)** Diagram of gene structure and mutation sites of *NtNPF6.13*. **(B)** Phenotype of *ntnpf6.13-1* mutant under salt stress compared with K326. **(C)** Root length of *ntnpf6.13-1* mutant and K326 under salt stress. The error bars and asterisks indicate the SD and statistical significance of three biological replicates (Student’s *t*-test; ** *P<* 0.01), respectively. **(D)** Hydroponic culture of *ntnpf6.13-1* mutant and K326. **(E)** Root chloride contents of K326 and *ntnpf6.13-1* mutant.

To further evaluate the role of NtNPF6.13 in chloride absorption and transportation, *NtNPF6.13* knockout lines were generated using CRISPR/Cas9-mediated genome-editing technology. Three mutants, *ntnpf6.13-1*, *ntnpf6.13-2* and *ntnpf6.13-3*, were generated in the K326 background (**Figure 7A** and **Supplementary Figure S4**). Sequencing revealed that, in the target region of the first exon, *ntnpf6.13-1* and *ntnpf6.13-2*harbored a 1-bp insertion, whereas *ntnpf6.13-3* had a 1-bp deletion, resulting in frame shift mutations of*NtNPF6.13* for all three mutants. By selfing and genotyping, we selected homozygous *ntnpf6.13-1* mutants lacking the Cas9 transgene for further analyses.

The growth of *ntnpf6.13-1* mutants was significantly suppressed under salt stress in comparison with K326 control plants (**Figure 7B**). Under the non-stress condition, almost no difference in plant growth was observed between the mutant and K326. Under salt stress (induced by 150mM NaCl treatment), the root length of the mutant was significantly shorter than that of K326 (**Figure 7C**). The Cl^-^ concentration in the roots was reduced by 36.1% in *ntnpf6.13-1* mutants compared with that of the K326 control plants (**Figure 7D, E**). Under treatment with NaCl (150 and 300 mM, respectively), the Cl^-^ concentration in the roots was also reduced by 24% and 15.5%, respectively, in the *ntnpf6.13-1* mutants (**Supplementary Figure S5A**). These results indicated that NtNPF6.13 might play an important role in Cl^-^ absorption in tobacco. Compared to K326 control plants, the chloride contents were significantly (p<0.01) increased in the leaves of *ntnpf6.13* mutant both before and after 150mM NaCl stress, while no significant difference was observed under 300mM NaCl stress (**Supplementary Figure S5B**). Even though the down-regulated chloride contents in root of *ntnpf6.13* was observed in comparison with K326, the chloride content did significantly increase in root of *ntnpf6.13* after NaCl stress. Therefore, it should be reasonable to conclude that NtNPF6.13 might affect the transportation of chloride from roots to shoots, but such effect would be neglectable small under higher Cl^-^ content in root.

## Discussion

In this study, 143 *NtNPF* genes in tobacco (K326) were identified and classified into eight groups consistent with the gene families of other species. As *NPF* gene families are characterized in an increasing number of plant species, the number of *NPF* members might be determined to be associated with ploidy. For example, hexaploidy wheat (*Triticum aestivum*) has the largest known *NPF* family (331 members) (Wang et al., 2020), and allotetraploid rapeseed (*Brassica napus*) has 193 *NPF* genes (Zhang et al., 2020). In contrast, diploid plants have dozens of *NPF* genes, such as Arabidopsis (53) (Krapp et al., 2014), rice (82) (Yang et al., 2020), apple (73) (Wang et al., 2018a) and spinach (57) (Wang et al., 2021).Tetraploid tobacco was determined to have 143 *NtNPF* genes in the present study. In addition, the phylogenetic classification of the *NtNPF* members was similar to other species accessed with the *NPF* genes classified into eight subfamilies. Compared with *AtNPF5*, which comprised the most members among *AtNPF* subfamilies, *NtNPF2* and *NtNPF5* were the largest subfamilies, with 25memberseach, among the eight *NtNPF* subfamilies. The intragenomic snyteny analysis revealed that 39 *NtNPF* genes formed syntenic blocks, indicating that the *NtNPF* gene family might have experienced whole-genome duplication and/or segmental duplications.

Almost 38% (55 genes) of the *NtNPF* genes were not mapped to tobacco chromosomes. The genome assembly used in this study was the latest version for *N. tabacum*, which was assembled by Edwards et al. (2017). Although it has been improved, anchorage of the tobacco genome to chromosomal locations was increased to 64%. It is expected that additional *NtNPF* genes will be mapped to chromosomes with further improvements in the tobacco genome assembly.

Considering previous *NPF* family analyses in different plant species, although the main focus has been nitrogen use efficiency (Wang et al., 2018a; Wang et al., 2020; Yang et al., 2020; Wang et al., 2021; Zhang et al., 2021; Zhao et al., 2021), the participation of certain *NPF* genes in chloride transport has been noted (Wen et al., 2017; Xiao et al., 2021).In the present study, we explored the *NtNPF* genes that contribute to chloride transportation in tobacco. The RNA-seq data for *NtNPF* genes under salt stress showed that most *NtNPF6* genes were down-regulated after salt stress exposure for 12 h. Ten NtNPF6s (NtNPF6.9-NtNPF6.18) that were homologous to AtNPF6.3 responded in the roots to salt stress (**Supplementary Figure S3**).In AtNPF6.3-like proteins, His356 is the structural element crucial for nitrate transportation (Wen and Kaiser, 2018). Mutation of this conserved His residue to Tyr conferred the ability to transport chloride (Xiao et al., 2021). Alignment of the conserved residues showed that six of the ten NtNPF6s, including NtNPF6.13, contained a Tyr residue at the position corresponding to AtNPF6.3:H356 (**Supplementary Table S5**), which indicated that these NtNPF6s might function in chloride metabolism. Recently, it was reported that the AtNPF6.3 subclade in rosids could be grouped into three haplotypes (A-, B-, C-type)based on four residues corresponding to T101, H356, T360 and F511 of AtNPF6.3 (Xiao et al., 2021). In addition, the haplotypes of ten NtNPF6s of AtNPF6.3 orthologs were analyzed (**Supplementary Figure S6** and **Supplementary Table S5**). NtNPF6.13 to NtNPF6.18 was unable to be classified to any subtype owing to the varying deficiencies among THTF residues. The other four AtNPF6.3 orthologs, NtNPF6.9 to NtNPF6.12, were all classified to the A-type (TYTF). It was also noted that NtNPF6.3 and 6.4 (AtNPF6.4 orthologs) and NtNPF6.5 to 6.8 (AtNPF6.2 orthologs) were responsive to salt stress in the leaves (**Figure 5** and **Supplementary Figure S7**).Whether these NtNPF6s play roles in leaf chloride metabolism requires additional experimental evidence. In addition to*NtNPF6* genes, other *NtNPF* genes were responsive to salt stress, such as *NtNPF7.11*, *NtNPF5.11* and *NtNPF4.21*. Previously, AtNPF7.3 and AtNPF7.2 were reported to play roles in chloride transportation (Li et al., 2016a). It would be promising to elucidate the function of NtNPF7.11in tobacco chloride metabolism.

In this study, we showed that *NtNPF6.13* may function in Cl^-^ uptake and transportation, but the exact molecular mechanism of NtNPF6.13 remains unknown. As ZmNPF6.4 and ZmNPF6.6 could transport both chloride and nitrate in maize, it was interesting to explore whether *NtNPF6.13* is involved in $NO_{3}^{-}$ transportation. As shown in **Supplementary Figure S8**, the root of *ntnpf6.13* mutant showed a significantly (p<0.01) down-regulated nitrate content only under normal condition (control), while in the leaves, the significantly decreased nitrate content of *ntnpf6.13* mutant was observed under both control and 300mM NaCl conditions. The lower nitrate content in *ntnpf6.13* mutant suggested that NtNPF6.13 did affect nitrate transport besides chloride in tobacco. That is, both nitrate and chloride could be the substrate of NtNPF6.13, and further studies are needed to identify their characters on affinity and efficiency of uptake and transport.

Meanwhile, Na^+^, brought in with Cl^-^, was also of interest and was determined. As shown in **Supplementary Figure S9**, *ntnpf6.13* mutant showed a significant (p<0.05) higher Na^+^ concentration in root under control, and a significant (p<0.05) higher Na^+^ concentration in leaves under salt treatment. Obviously, the changes of Na^+^ and Cl^-^ were converse at roots. Such contrast changes upon cation and anion were also observed on other transportation proteins (such as NtSLAH) related transgenic lines (unpublished data). All of these indicated the complexity of ion transport mechanism, and need to be further studied and clarified.

As a stress signal of higher amount of NaCl, *ntnpf6.13* mutant responded this salt stress with changes on gene expression level, Na^+^ content, Cl^-^ content, $NO_{3}^{-}$ content, root length, etc. In addition, several light responsive elements such as Box 4, GT1-motif and TCT motif, were detected in the *NtNPF6.13* promoter (**Supplementary Table S3**), which indicated NtNPF6.13 might also be regulated by light signal.

## Conclusion

A total of 143 *NtNPFs* were identified and the phylogenetic relationships, chromosomal distribution and conserved domain structure were analyzed. RNA-seq data showed that the expression level of most *NtNPF6* genes (10 in roots and 6 in leaves) was reduced after treatment with 300mM NaCl for 12h. The gene*NtNPF6.13* was responsive to salt stress in the roots and was localized to the plasma membrane. After knockout by CRISPR/Cas9, *ntnpf6.13* mutants showed a significantly reduced Cl^-^ content in the roots compared with that of the control K326. In addition, the root length of the mutants was significantly shorter than that of K326 after exposure to salt stress. These results showed that NtNPF6.13 might be involved in the absorption of Cl^-^ in tobacco. Further studies are needed to explore the function of other NtNPF6s that responded to salt stress, and their molecular mechanisms in chloride absorption and transportation.

## Data availability statement

The datasets presented in this study can be found in online repositories. The names of the repository/repositories and accession number(s) can be found below: https://www.ncbi.nlm.nih.gov/, PRJNA827645.

## Author contributions

HZ and HnZ conceived the experiments. ZH drafted the manuscript. ZL analyzed the RNA-seq data. GX, GB, PZ and NZ participated in the main experiments in this work, with assistance from QZ, QC and PL. LJ and HNZ contributed the revise of the manuscript. All authors contributed to the article and approved the submitted version.

## Funding

This work was supported by grants from the CNTC Research Program [grant no. 110202001018(JY-01)] and YNTC Research Program (grant no. 2021530000241016).

## Acknowledgments

We thank Towin Biotechnology (www.towinbio.com) for providing the pCS1300 vector. We also thank Charlesworth Author Services (https://www.cwauthors.com.cn) for language editing.

## Conflict of interest

Author HZ, ZL, GX, PZ, NZ, QZ, QC, PL, LJ, HnZ are employed by Zhengzhou Tobacco Research Institute of CNTC. This study received funding from CNTC and YNTC. The funder was not involved in the study design, collection, analysis, interpretation of data, the writing of this article or the decision to submit it for publication.

The remaining authors declare that the research was conducted in the absence of any commercial or financial relationships that could be construed as a potential conflict of interest.

## Publisher’s note

All claims expressed in this article are solely those of the authors and do not necessarily represent those of their affiliated organizations, or those of the publisher, the editors and the reviewers. Any product that may be evaluated in this article, or claim that may be made by its manufacturer, is not guaranteed or endorsed by the publisher.

## Supplementary material

Supplementary Table 1 Sequences of *NtNPF6*-specificqPCR primers.

Supplementary Table 2 Characterization of the *NtNPF* family in tobacco.

Supplementary Table 3
*Cis*-acting elements of *NtNPF* genes.

Supplementary Table 4 Expression analysis of *NtNPF* genes responsive to salt stress according to RNA-seq data.

Supplementary Table 5 Haplotype of AtNPF6.3 subclade.

Supplementary Figure 1 Numbers of different *cis*-acting elements in *NtNPF* genes.

Supplementary Figure 2 Expression profiles of *NtNPF* genes under salt stress.

Supplementary Figure 3 Expression pattern analysis of all *NtNPF* genes under salt stress.

Supplementary Figure 4 Schematic diagram of gene structure and mutation sites of *NtNPF6.13*.

Supplementary Figure 5 Cl^−^ contents in root **(A)** and leave **(B)** of K326 and *ntnpf6.13-1* mutant under salt stress.

Supplementary Figure 6 AtNPF6.3 subclade in different plant species.

Supplementary Figure 7 Phylogenetic analysis of NPF proteins.

Supplementary Figure 8

$NO_{3}^{-}$ contents in root **(A)** and leave **(B)** of K326 and *ntnpf6.13-1* mutant under salt stress.

Supplementary Figure 9 Na^+^ contents in root **(A)** and leave **(B)** of K326 and *ntnpf6.13-1* mutant under salt stress.
