# Supplementary figures and images for: Genome-wide identification and characterization of NPF family reveals NtNPF6.13 involving in salt stress in Nicotiana tabacum
Source: Front Plant Sci. 2022 Oct 13;13:999403. doi: 10.3389/fpls.2022.999403 (PMC9608447; doi:10.3389/fpls.2022.999403)

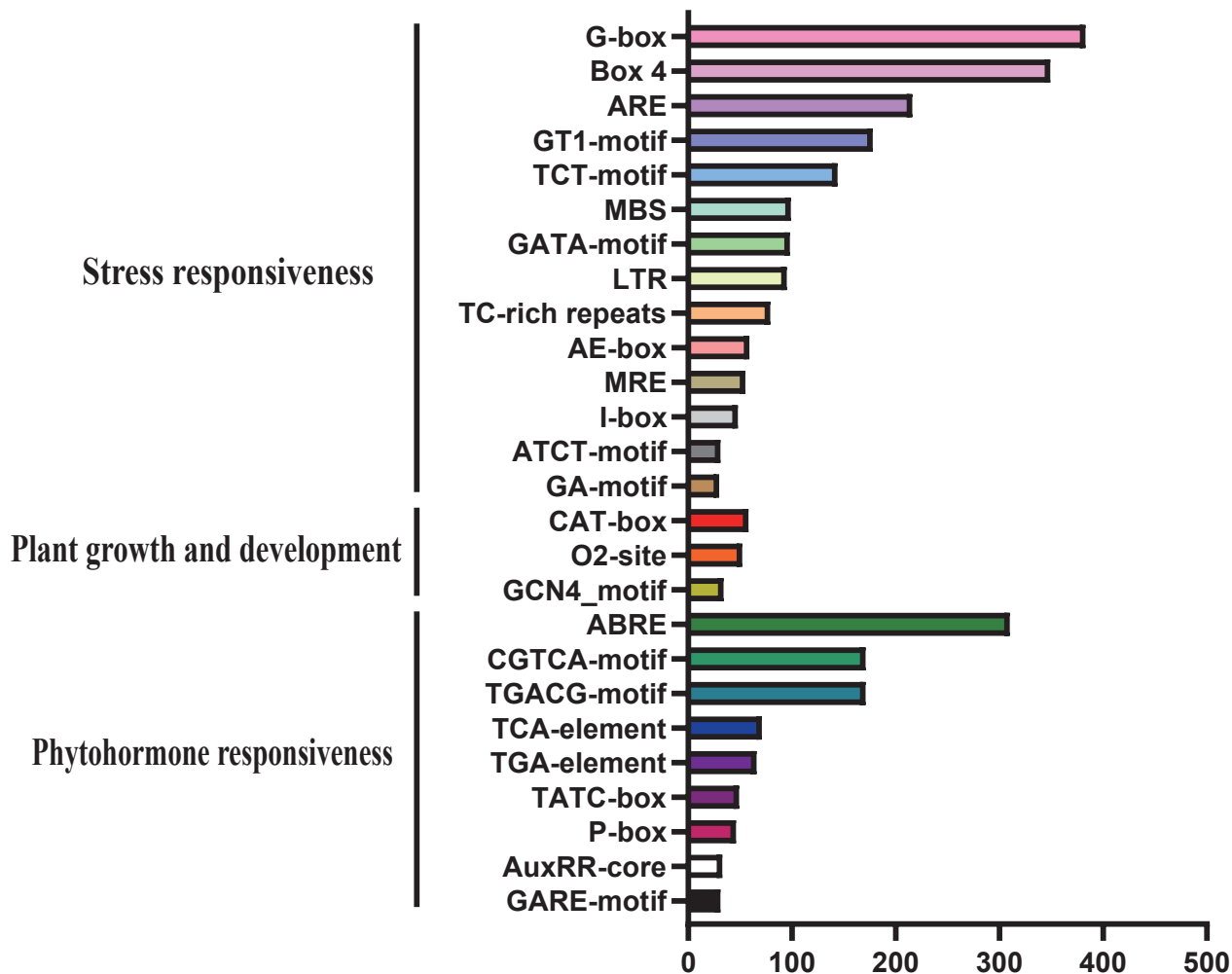

Supplement: Supplementary Figure 1 — Numbers of different cis-acting elements in NtNPF genes. [file Image_1.pdf]

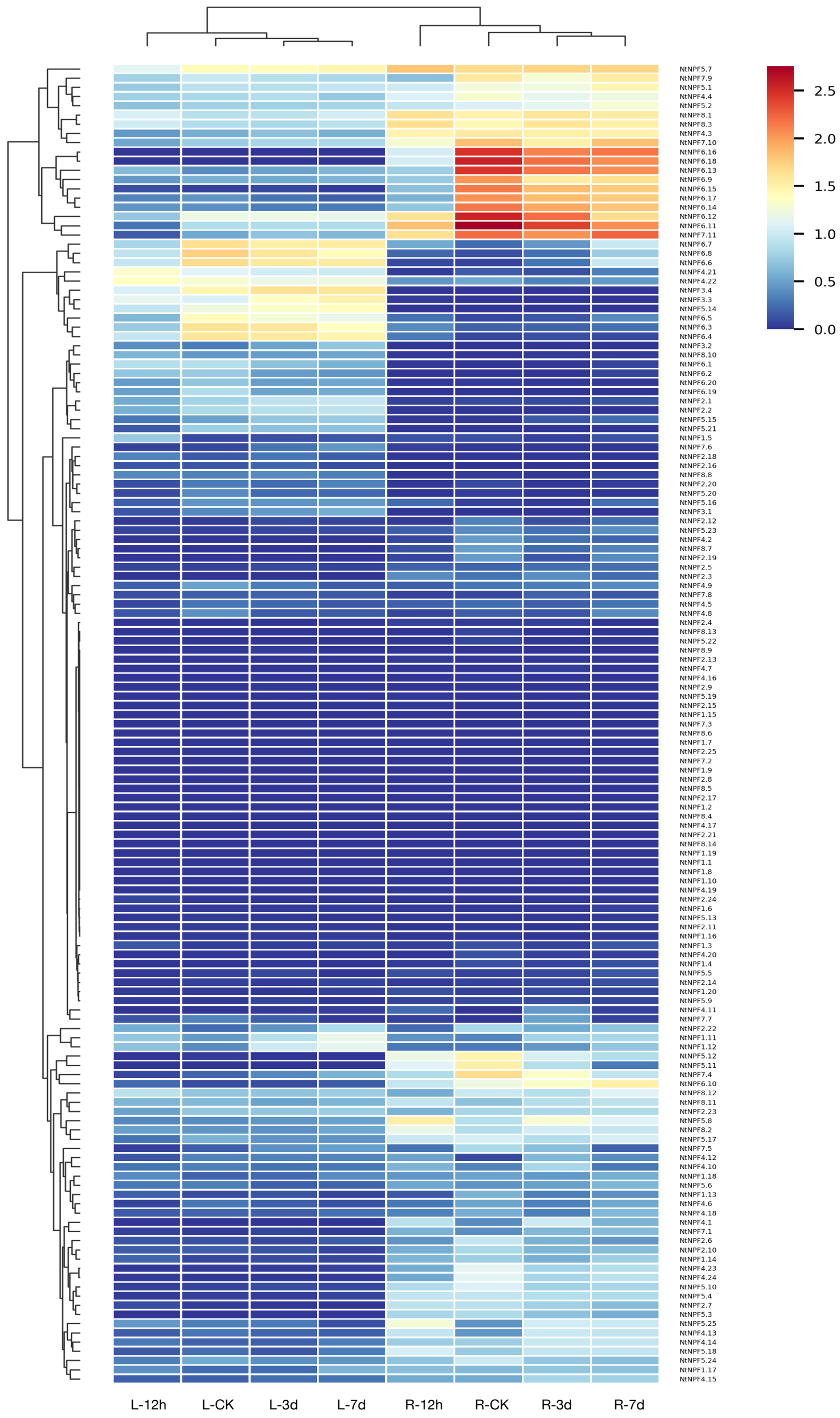

Supplement: Supplementary Figure 2 — Expression profiles of NtNPF genes under salt stress. [file Image_2.pdf]

***NtNPF6.3***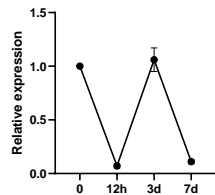***NtNPF6.4***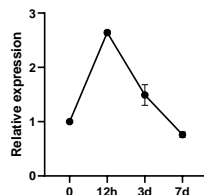***NtNPF6.6***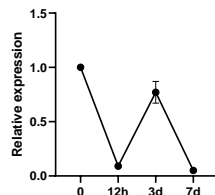***NtNPF6.7***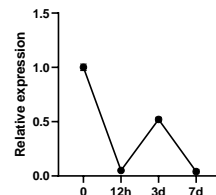***NtNPF6.8***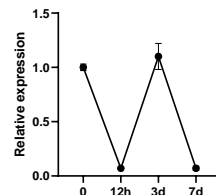***NtNPF6.9***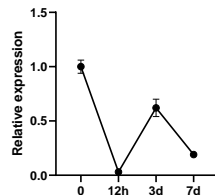***NtNPF6.10***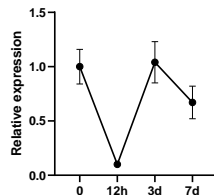***NtNPF6.11***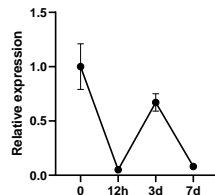***NtNPF6.12***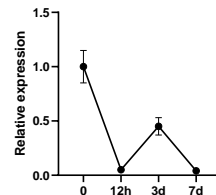***NtNPF6.13***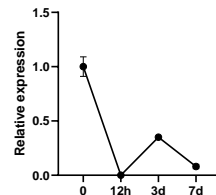***NtNPF6.14***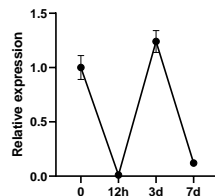***NtNPF6.15***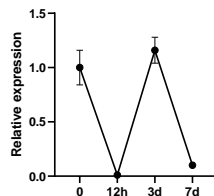***NtNPF6.16***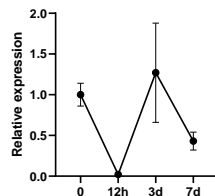***NtNPF6.17***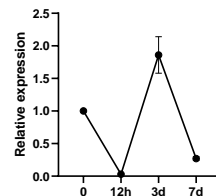***NtNPF6.18***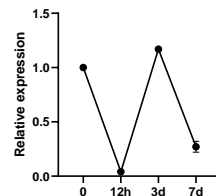

Supplement: Supplementary Figure 3 — Expression pattern analysis of all NtNPF genes under salt stress. [file Image_3.pdf]

**A.**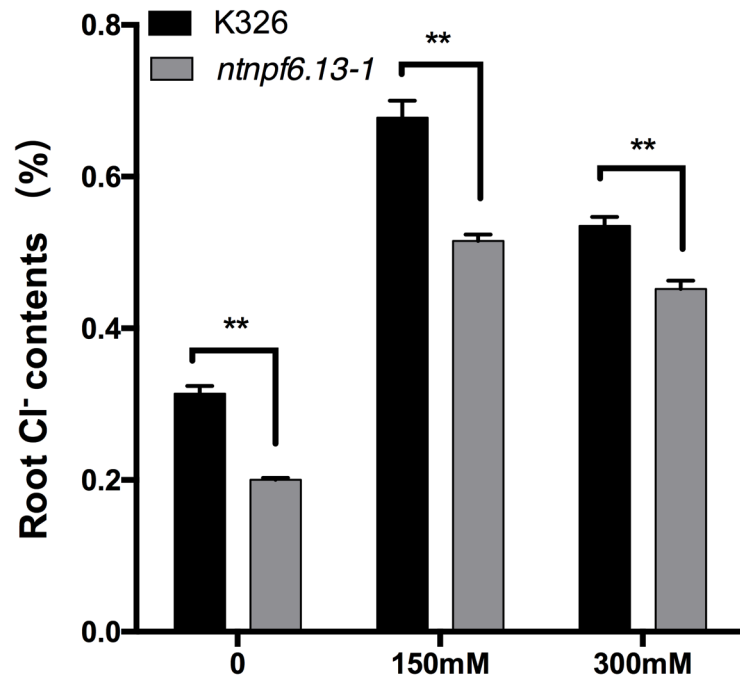**B.**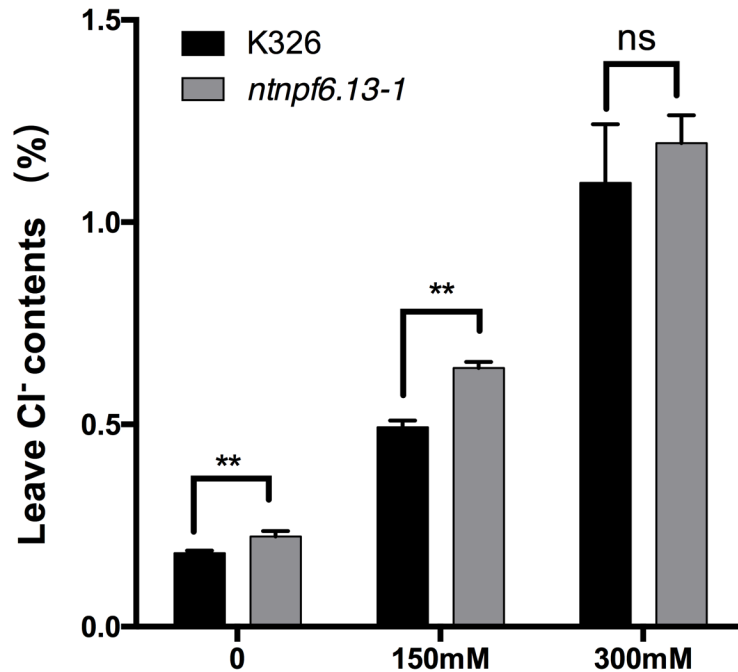

Supplement: Supplementary Figure 5 — Cl− contents in root (A) and leave (B) of K326 and ntnpf6.13-1 mutant under salt stress. [file Image_5.pdf]

### Colored ranges

- A-type
- B-type
- C-type

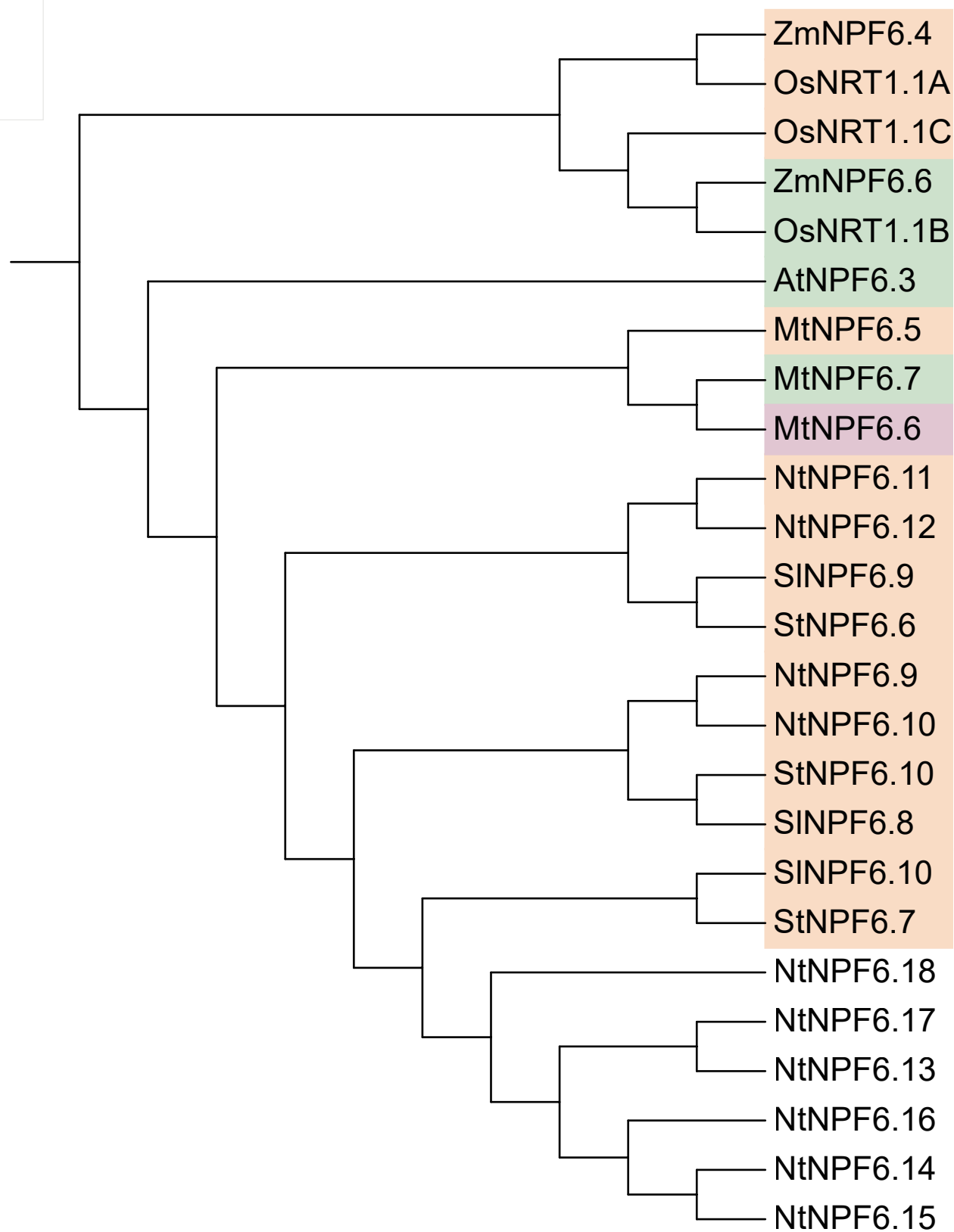

Supplement: Supplementary Figure 6 — AtNPF6.3 subclade in different plant species. [file Image_6.pdf]

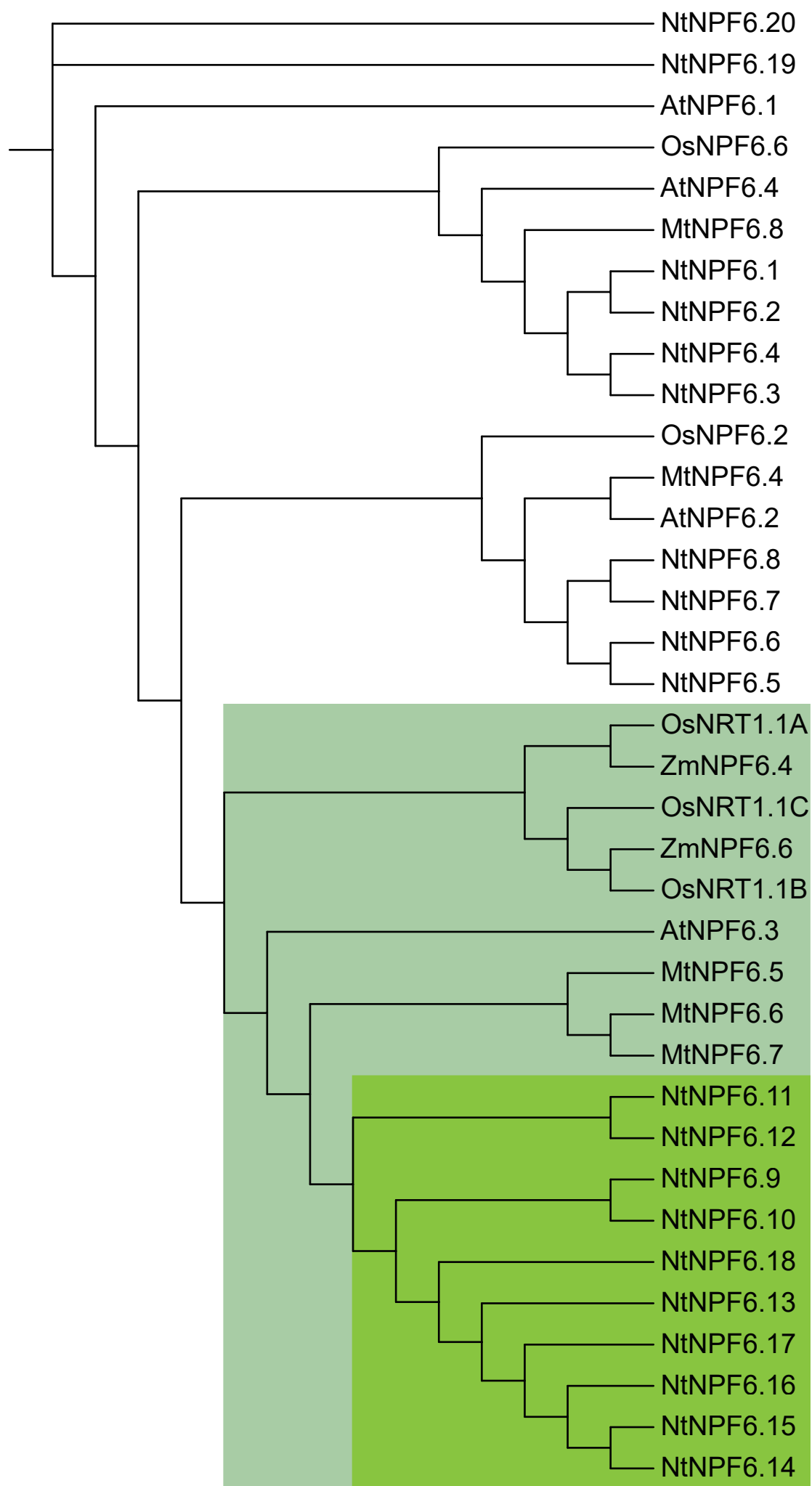

Supplement: Supplementary Figure 7 — Phylogenetic analysis of NPF proteins. [file Image_7.pdf]

**A.**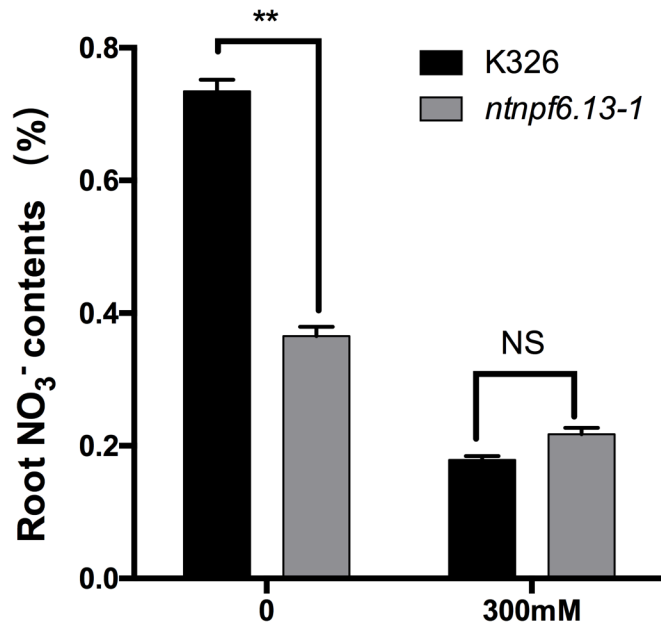**B.**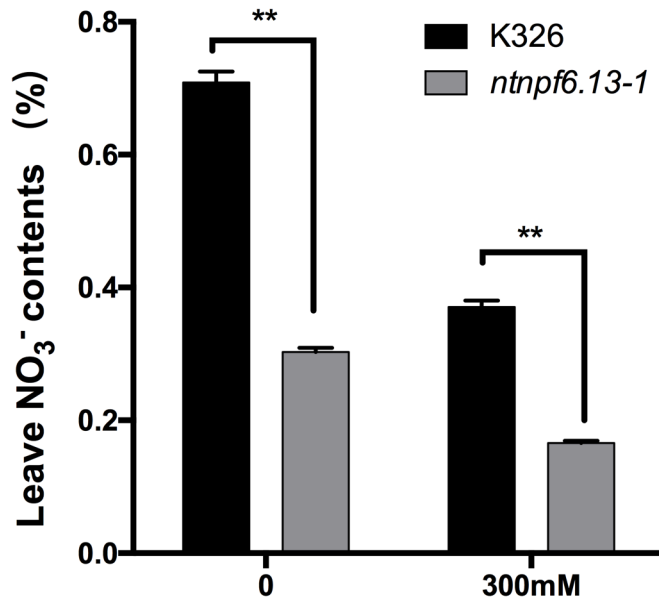

Supplement: Supplementary Figure 8 — NO3− contents in root (A) and leave (B) of K326 and ntnpf6.13-1 mutant under salt stress. [file Image_8.pdf]

**A.**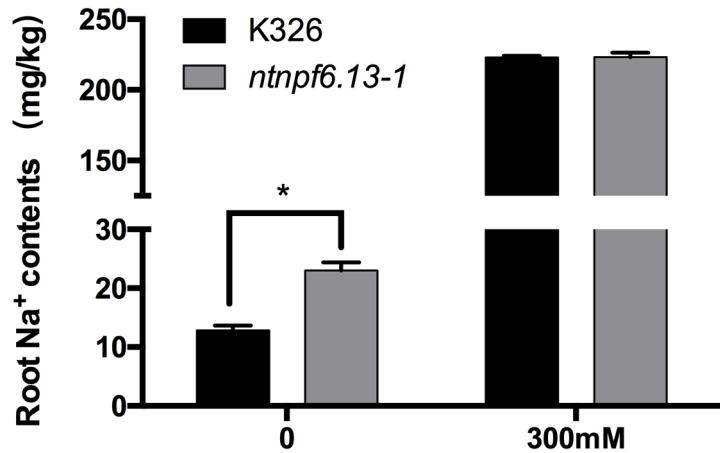**B.**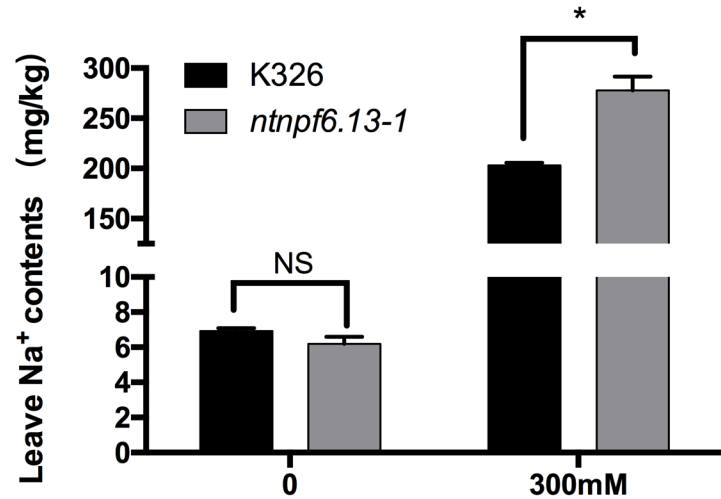

Supplement: Supplementary Figure 9 — Na+ contents in root (A) and leave (B) of K326 and ntnpf6.13-1 mutant under salt stress. [file Image_9.pdf]
