# Supplementary material for: Genome-wide identification and characterization of NPF family reveals NtNPF6.13 involving in salt stress in Nicotiana tabacum
Source: Front Plant Sci. 2022 Oct 13;13:999403. doi: 10.3389/fpls.2022.999403 (PMC9608447; doi:10.3389/fpls.2022.999403)
Supplement: Supplementary Figure 4 — Schematic diagram of gene structure and mutation sites of NtNPF6.13. [file Image_4.pdf]

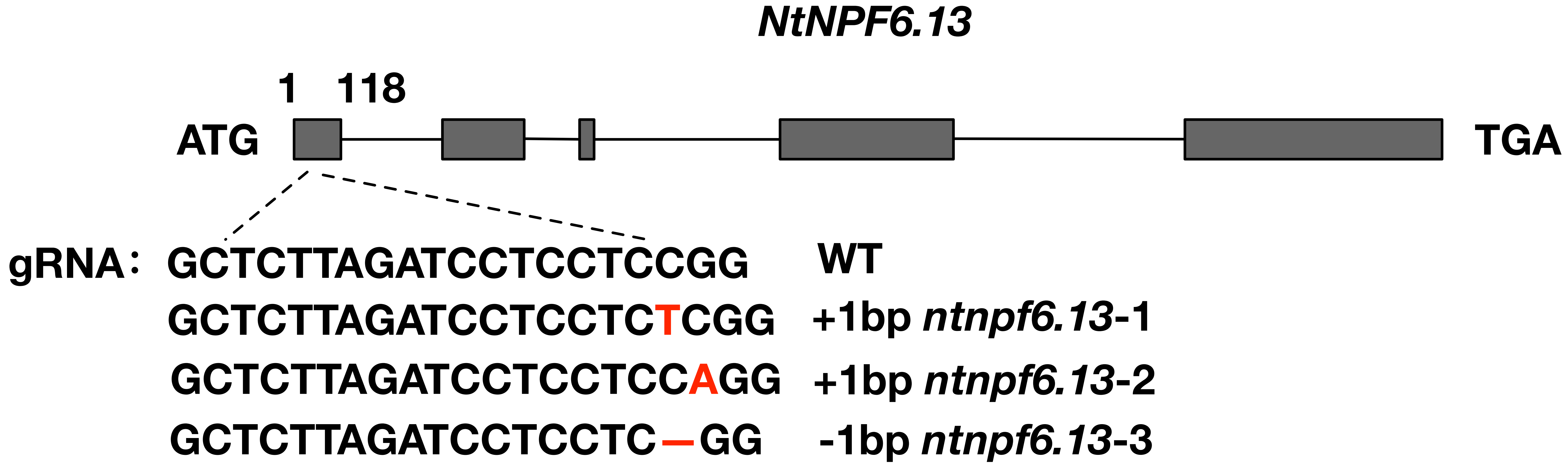
NtNPF6.13
1
118
ATG
TGA
gRNA：GCTCTTAGATCCTCCTCCGG
WT
GCTCTTAGATCCTCCTCTCGG
+1bp ntnpf6.13-1
GCTCTTAGATCCTCCTCCAGG
+1bp ntnpf6.13-2
GCTCTTAGATCCTCCTC—GG
-1bp ntnpf6.13-3
